# Polyglutamine Tract Expansion Increases S-Nitrosylation of Huntingtin and Ataxin-1

**DOI:** 10.1371/journal.pone.0163359

**Published:** 2016-09-22

**Authors:** Chun-Lun Ni, Divya Seth, Fabio Vasconcelos Fonseca, Liwen Wang, Tsan Sam Xiao, Phillip Gruber, Man-Sun Sy, Jonathan S. Stamler, Alan M. Tartakoff

**Affiliations:** 1 Cell Biology Program, Department of Molecular Biology and Microbiology, Case Western Reserve University, Cleveland, OH, 44106, United States of America; 2 Institute for Transformative Molecular Medicine, Case Western Reserve University, Cleveland, OH, 44106, United States of America; 3 Center for Proteomics and Bioinformatics, Case Western Reserve University, Cleveland, OH, 44106, United States of America; 4 Department of Pathology, Case Western Reserve University, Cleveland, OH, 44106, United States of America; 5 Department of Biochemistry, School of Medicine, Case Western Reserve University, Cleveland, OH, 44106, United States of America; Emory University, UNITED STATES

## Abstract

Expansion of the polyglutamine (polyQ) tract in the huntingtin (Htt) protein causes Huntington’s disease (HD), a fatal inherited movement disorder linked to neurodegeneration in the striatum and cortex. S-nitrosylation and S-acylation of cysteine residues regulate many functions of cytosolic proteins. We therefore used a resin-assisted capture approach to identify these modifications in Htt. In contrast to many proteins that have only a single S-nitrosylation or S-acylation site, we identified sites along much of the length of Htt. Moreover, analysis of cells expressing full-length Htt or a large N-terminal fragment of Htt shows that polyQ expansion strongly increases Htt S-nitrosylation. This effect appears to be general since it is also observed in Ataxin-1, which causes spinocerebellar ataxia type 1 (SCA1) when its polyQ tract is expanded. Overexpression of nitric oxide synthase increases the S-nitrosylation of normal Htt and the frequency of conspicuous juxtanuclear inclusions of Htt N-terminal fragments in transfected cells. Taken together with the evidence that S-nitrosylation of Htt is widespread and parallels polyQ expansion, these subcellular changes show that S-nitrosylation affects the biology of this protein *in vivo*.

## Introduction

**Fig 1:**
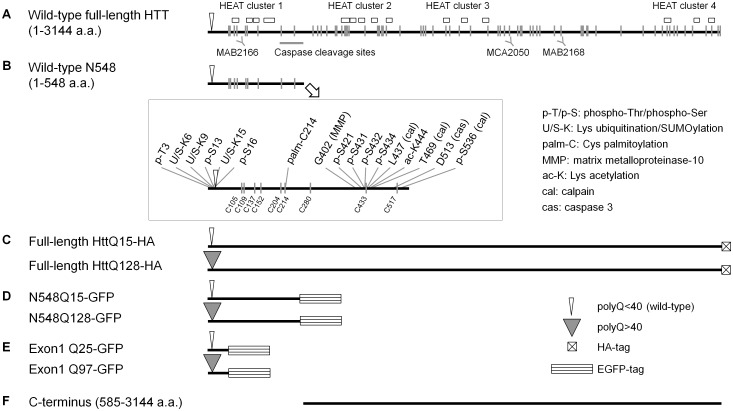
Full-length Htt and Htt fragments. (A) Wild-type (wt) full-length Htt. The vertical lines indicates all 70 Cys residues. The horizontal open boxes indicate HEAT repeat motif clusters (Tartari, M. *et al*., *Mol Biol Evol*. 2008). The horizontal grey bar labels caspase cleavage sites. Anti-Htt antibodies (MAB2166, MCA2050, and MAB2168) and their binding sites are labeled. (B) Htt N548 fragment (1–548 a.a.) and its posttranslational modifications. The inset is an enlargement of this region. Cysteine residue numbers are indicated. (C) Full-length (FL) Htt constructs for transient expression. (D) Htt N-terminal 1–548 a.a. fragments that approximate proteolytic products of full-length Htt. (E) Constructs for expressing Htt exon1 coding region (1–90 a.a.). (F) C-terminal construct (585–3144 a.a.).

Huntington’s disease (HD) is an inherited movement disorder characterized by progressive degeneration of medium spiny neurons of the striatum, as well as other neuronal cell types. The pathogenic mutations of the HTT gene are (CAG) trinucleotide repeats that exceed 40. The (CAG) repeats encode a polyglutamine tract (polyQ) near the N-terminus of the Htt protein (Fig 1A) [1–6].

Full length Htt is conserved among vertebrates and chordates and is widely expressed in nucleated cells [7–9]. Expression of Htt is essential for embryogenesis in mouse models [10]; however, the function(s) of Htt are not well understood. Htt is found primarily in the cytoplasm. Nevertheless, in certain cell types a fragment of Htt enters the nucleus [11]. In the cytosol, Htt is associated with vesicles, the Golgi complex, mitochondria and the mitotic spindle [12–19]. Expansion of the polyQ tract of Htt affects protein-protein interactions, reduces levels of brain-derived neurotropic factor, and inhibits microtubule motor function [20–25].

Many proteins interact with both normal and polyQ-expanded Htt. These include proteins implicated in 14-3-3 signaling, microtubule-based transport, proteostasis, and fission of mitochondria [26]. Several of these interactions have been mapped to the N-terminal segment of Htt and are influenced by polyQ expansion [27]. Large amounts of polyQ-expanded forms of Htt accumulate in the cytoplasm, where they form dynamic “aggregates” [5, 28]. However, it remains controversial as to whether aggregation of Htt accounts for its toxicity. Studies of cultured cells as well as the mouse brain show that N-terminal fragments of polyQ-Htt are especially toxic. Moreover, the N-terminal fragment derived from cleavage at D586 is required for the pathogenesis of HD in a mouse model [29]. Administration of an inhibitory peptide containing the sequence around D586 protects a HD mouse model from pathogenesis [30].

In addition to proteolytic cleavage, Htt undergoes multiple posttranslational modifications [31–35]. For example, more than 10 phosphorylation sites have been identified and their relation to polyQ expansion is complex and residue-dependent. In addition, polyQ expansion increases Htt ubiquitination and SUMOylation [36–38]. One study suggests that polyQ expansion reduces Htt S-palmitoylation [15].

Cysteine modifications, such as S-nitrosylation and S-acylation regulate the functions, physical properties and localization of many proteins [39–44], including protein-protein interactions, cell signaling, and enzyme activities [45]. S-nitrosylation typically requires nitric oxide (NO) that is produced by nitric oxide synthase (NOS) [46] and corresponding transferase(s) that are only incompletely characterized. The mammalian genome encodes three NOS isoforms: neuronal NOS (nNOS/NOS1), endothelial NOS (eNOS/NOS3), and inducible NOS (iNOS/NOS2) [47]. nNOS and eNOS are constitutively expressed in neurons and in the vascular endothelium, respectively [47], as well as in many other cell types. iNOS is induced in most cell types upon inflammation. Thus, virtually all cells can make NO. The detailed mechanism of NO transfer from NOS to target proteins is elusive. In some cases, proteins that form a complex with NOS can direct S-nitrosylation to specific cysteine residues [48].

Alterations of protein S-nitrosylation and S-acylation have been reported in several neurodegenerative diseases, including HD [49]. However, few sites have been identified and the relationship between polyQ expansion and Htt cysteine modification has not been investigated. In this study we provide evidence that there is a strong link between the levels of Htt polyQ expansion and the levels of its S-nitrosylation and S-acylation. We have identified multiple sites of Htt S-nitrosylation and S-acylation. Many of these sites are close to sites that are phosphorylated or acetylated (Fig 1B) [32]. Similarly, S-nitrosylation increases upon expansion of the polyQ tract of Ataxin-1, which is the cause of spinocerebellar ataxia type 1 (SCA1) [50]. Therefore, polyQ-dependent cysteine modifications could be broadly significant for neurodegenerative diseases.

## Materials and Methods

### Reagents

Collagen (C5533), HEPES (H4034), EDTA (E5134), Diethylene triamine pentaacetic acid (DTPA; D6518), Neocuproine hydrochloride (72090), SDS (L6026), S-Methyl methanethiosulfonate (MMTS; 64306), N-Ethylmaleimide (NEM; 04260), N-Methylmaleimide (NMM; 389412), Iodoacetamide (I1149), Sodium L-ascorbate (11140), Hydroxylamine hydrochloride (55459), Anti-FLAG antibody (F1978; 1:2000 for the western), Anti-actin antibody (A1978; 1:2000 for the Western), poly-L-lysine solution (P4707), and histology mounting medium (with DAPI, F6057) were from Sigma. DMEM medium (11965092), F12K medium (21127022), Horse serum (26050088), FBS (26140079), and HBSS buffer (14025076) were from Gibco. Protease inhibitor cocktail (05892791001) and Protein G-agarose (11719416001) were from Roche. Anti-NOS1 (H-299, sc-8309; 1:1000 for the Western), anti-NOS2 (H-174, sc8310; 1:1000 for the Western), and anti-NOS3 (C-20, sc654; 1:1000 for the Western) were from Santa Cruz Biotechnology. Anti-Htt (N-terminal, clone 1HU4C8; MAB2166; 1:2500 for the Western) and Anti-Htt (C-terminal, clone HU-2E8; MAB2168; 1:2500 for the Western) were from EMD Millipore. SDS-Out SDS Precipitation kit (20308) and Imperial protein stain (24615) were from Thermo. BCA assay (23225) was from Pierce. Anti-Htt (C-terminal, clone HDB4E10; MCA2050; 1:2500 for the Western) was from AbD Serotec. Immun-Star AP chemiluminescence (1705010) was from Bio-rad. Polyclonal anti-GFP (for immunoprecipitation; ab290) was from Abcam. Monoclonal anti-GFP (for Western; 632381; 1:5000 for the Western) was from Clontech. Thiopropyl Sepharose 6B (17042001) was from GE. MS grade trypsin (V5111) was from Promega. Lipofectamine 3000 (L3000015) was from Invitrogen. Ponasterone A (P-1083) was from A.G. Scientific. NP40 (19628) was from USB.

### Mammalian cell culture and recombinant protein expression

Cells were grown at 37°C, with 5% CO_2_. HEK293T (gift from Dr. H-Y. Kao, CWRU) and COS7 (gift from Dr. M. Weiss, CWRU) cells were grown in DMEM with 5% FBS. Recombinant proteins were expressed in these cell lines by using Lipofectamine 3000, following the instructions of the manufacturer. Cells in a well of 6 well plate was transfected with 2.5 μg. Cells were harvested 1–2 days after transfection. No obvious growth reduction or toxicity was observed in our cell models expressing Htt N548 +/- polyQ expansion over a week.

PC12 cells were grown in F12K medium with 2.5% FBS and 15% horse serum. Tissue culture plates were coated with collagen before use. For this purpose, a stock of 1 mg/mL collagen in HBSS buffer/0.25% acetic acid was diluted to 0.2 mg/mL in HBSS buffer. Plates were coated for 20 min, the liquid was aspirated and the plates were then air-dried overnight. PC12 cells expressing inducible full-length Htt constructs (HttQ23 and HttQ73) were the kind gift from Dr. X. Qi (CWRU). Recombinant HttQ23 and HttQ73 were induced by adding ponasterone A (5 μM) for two days.

After harvesting by scraping cells in the presence of PBS including the chelator, DTPA (100 μM), cell pellets were washed twice with PBS/DTPA and snap frozen at -80°C.

### Mouse tissues

Tissue samples were from a B6 mice (3 months), originating from the Jackson Laboratory. Tissue homogenates were prepared in the buffer containing protease inhibitor cocktail that is also used for SNO-RAC/acyl-RAC (see below). All experimental procedures conducted upon live animals were first approved by the Institutional Animal Care and Use Committee of CWRU (Case Western Reserve University) School of Medicine and were conducted in accordance with the National Institutes of Health Guide for the Care and Use of Laboratory Animals. CWRU is a PHS-assured institution (Assurance # A-3145-01) and the institution is fully accredited by AAALAC (Association for Assessment and Accreditation of Laboratory Animal Care International).

### Plasmids used in this study

Htt constructs are shown in Fig 1. Full-length Htt and N-terminal 548 a.a. constructs (pCINeoHttFL.15Q.wt.HA, pCINeoHttFL.128Q.wt.HA, pCINeoHtt1955.15Q.wt.GFP, pCINeoHtt1955.128Q.wt.GFP, pCINeoHtt1955.15Q.C214S.GFP, and pCINeoHtt1955.128Q.C214S.GFP) and Htt C-terminus (585–3144) construct were the kind gifts from Dr. M. Hayden (University of British Columbia). The Htt exon1 constructs (pcDNA3.1 HttEx1-25Q-EGFP and pcDNA3.1 HttEx1-97Q-EGFP) were gifts from Dr. W. Yang (University of California, Los Angeles). Ataxin-1 constructs (pcDNA1 Flag ATXN1 [30Q] and pcDNA1 Flag ATXN1 [85Q]) were the gifts from Dr. Orr (University of Minnesota). Generation of nitric oxide synthase constructs (pCDNA3-nNOS, pCDNA3-eNOS, and pCDNA3-iNOS) was described in the previous study [51].

### Site-directed mutagenesis

To create the C204S mutant, the Htt N-terminal 548 a.a. constructs were used as template. Site-directed mutagenesis was done using a kit (Agilent Technologies, 200519). The C204S forward primer was:

5’-cggcctcagaaaagcaggccttacctggtgaac-3’.

The C204S reverse primer was:

5’- gttcaccaggtaaggcctgcttttctgaggccg-3’.

### Detection of protein S-nitrosylation and S-acylation by resin-assisted capture (RAC)

Resin-assisted capture of S-nitrosylated proteins (SNO-RAC) or S-acylated proteins (acyl-RAC) was described in previous studies [52, 53]. Cells were lysed on ice in HENS buffer (HEPES 100mM, EDTA 5 mM, Neocuproine 0.1%, and SDS 1%; pH8.0) containing NP40 1%, MMTS 0.1%, and a protease inhibitor cocktail. The extracts were then incubated in blocking buffer (HEPES 100mM, EDTA 5 mM, Neocuproine 0.1%, SDS 2.5%, and MMTS 0.1%; pH8.0) at 50°C for 20 min. This step blocks free thio-groups so that later addition of thiopropyl sepharose 6B beads will interact exclusively with the thio-groups resulting from selective reduction of S-nitrosylated or S-acylated residues, using ascorbate or hydroxylamine, respectively. After blocking, the samples were precipitated with acetone at -80°C for 15 min.

Protein pellets were washed with 70% acetone (4°C) three times and then resuspended in the binding buffer (HEPES 100mM, EDTA 5 mM, Neocuproine 0.1%, SDS 1%, and protease inhibitor cocktail; pH8.0). Protein was quantitated using a BCA assay.

For selective capture, the binding mixture contained Thiopropyl Sepharose 6B beads and the appropriate specific reducing reagent. For SNO-RAC, S-nitrosylated Cys residues were reduced by adding ascorbic acid (50 mM) so that beads covalently bound to them. For acyl-RAC, hydroxylamine (200 mM) was used to reduce S-acylated residues. A “buffer control” (no reducing reagent) was performed in parallel to detect any non-specific binding. Binding was performed on a shaker in the dark at room temperature for 3 hrs. The beads were then washed four times with HENS buffer and two times with 10% HEN/1% SDS buffer.

After elimination of excess liquid, protein was eluted from the beads using SDS sample buffer with β-mercaptoethanol. After 20 min incubation at room temperature and 4 min at 90°C, the eluate was collected and the samples were run in 7.5% gel for SDS-PAGE and analyzed by Western blotting. Quantification of images was with ImageJ.

### Detection of S-nitrosylation and S-acylation sites by LC-MS/MS

Free thiol-groups of proteins in cell extracts were first blocked with NEM, followed by acetone precipitation and resuspension in HENS buffer containing a protease inhibitor cocktail. Samples were then divided into two equal parts. One part was incubated with ascorbate (50 mM) and IAA (100 mM) for 1 hr to reduce S-nitrosylated residues and label them. The other part was incubated with hydroxylamine (200 mM) and IAA (100 mM) for 1 hr to reduce S-acylated residues and label them.

When completing the labeling reaction, protein was again acetone-precipitated and then resuspended in IP buffer (Tris-HCl 20 mM, NaCl 137 mM, EDTA 2 mM, NP-40 1%, glycerol 10%, and protease inhibitor cocktail, pH 8.0) plus SDS 1%. After resuspension, SDS was eliminated using a SDS precipitation kit (Thermo, 20308). Samples were pre-cleared by incubating with a protein G-agarose slurry (50%, 20 μL) in the cold for 1 hr. The supernatant was collected and incubated with antibody (~30 μg) in the cold for 1 hr. EGFP-tagged Htt N548 fragments were immunoprecipitated by anti-GFP (ab290; 1:50 for immunoprecipitation) and full-length Htt proteins by anti-Htt (C-terminal, MCA2050; 1:50 for immunoprecipitation). The bead slurry (50%, 50 μL) was then added for an additional 3 hr. After washing three times with PBS, protein was eluted by incubating with SDS sample buffer containing β-mercaptoethanol. After 20 min incubation at the room temperature and 4 min at 90°C, the eluate was recovered and proteins in samples were resolved by SDS-PAGE on gradient gels. For the high molecular weight (HMW) species, a group of samples were incubated at 37°C to determine these HMW species were not induced by heat.

After staining with Imperial Protein Stain (Thermo), protein bands were excised, destained in a mixture of 50% 100 mM ammonium biocarbonate and 50% acetonitrile and reduced with DTT, followed by excess NMM to reduce disulfides and to block the resulting free cysteine residues. After washing with 100 mM ammonium carbonate and 100% acetonitrile—alternately—three times, gel bands were spin-dried. A sequencing grade trypsin (Promega) solution (500 ng in 50 μL 100 mM ammonium bicarbonate) was incubated with the dried bands overnight at 37°C.

Htt tryptic peptides were subjected to LC-MS/MS analysis using an Orbitrap Elite hybrid mass spectrometer (Thermo Scientific). Nano-reverse phase liquid chromatography separations were performed on a UPLC (Waters, Milford, MA) directly connected to a nanospray emitter (10 μm, New Objectives). LC separation was conducted by using mobile phases A (0.1% formic acid in water) and B (100% acetonitrile) with a 90 min linear gradient starting with 1% B and increasing to 40% phase B. All data were acquired in positive ion modes. Full MS scans (m/z 300–2000) were followed by MS2 scans of the ten most abundant peptide ions at normalized collision energy of 35%. High mass accuracy FT/MS was performed to detect precursor ions (resolution, 60,000; mass accuracy, 5 ppm).

### MS data analysis

Bioinformatics software MassMatrix (2.4.2, Feb 22, 2012) [54] was used to search tandem MS data against a database containing the Htt sequence and decoy sequences with reversed sequence of this protein. We targeted trypsin-digested peptides containing cysteine residues. The M/Z shift of targeted peptides due to cysteine modifications [iodoacetamide (IAA), N-ethyl-maleimide (NEM), and N-methyl-maleimide (NMM)], were selected for detection by mass spectrometry. According to the sample preparation described above, NEM-labeled cysteines were unmodified, IAA-labeled cysteine residues were S-nitrosylated/S-acylated, and the remaining cysteine residues with other modifications such as disulfide bond were labeled by NMM after DTT reduction.

Precursor ions were searched with 10 ppm mass accuracy and product ions were searched with 1 Da mass accuracy. Peptide identifications with or without modifications were determined with PP scores greater than 5.0 and PPtage scores greater than 2.0. All of the detected modified peptides mass spectra were manually checked. LC-MS/MS and MS analysis were performed in the Center for Proteomics and Bioinformatics, Case Western Reserve University.

### Fluorescent microscopy study on Htt N548 inclusions

HEK293T cells were grown in 12 well tissue culture plate with poly-lysine-coated cover slips. Htt N548-EGFP construct mixed with empty vector or NOS construct in 1:1 ratio (ng) was used to transfect HEK293T cells by Lipofectamine 3000 (L3000015). After two days expression, cells were fixed by 4% formaldehyde at room temperature in dark for 10 minutes. Then rinsed the samples with PBS for three times. The mounting medium (with DAPI) was then applied to the sample incubation at the room temperature in dark for 5 minutes, followed by sealing with nail polish. The EGFP-positive cells were counted to calculate the percentage of cells with N548-EGFP inclusion(s). Fluorescent signals from DAPI and EGFP were acquired by LEICA DMI4000 B microscopy/LEICA DFC345 FX camera. To obtain high resolution image, DeltaVision RT wide-field fluorescence imaging system (Applied Precision, Inc) and CCD digital camera (Photometrics CoolSnap HQ) were used. To remove out-of-focus light, deconvolution software (Applied Precision, Inc) was applied.

### Htt HEAT repeat simulation

The MMM server (http://manaslu.aecom.yu.edu/MMM) [55] was used to create the molecular models for the A, B, C and D clusters of HEAT repeats (Tartari *et al*., 2008) [9] using the crystal structure of the first eight HEAT repeats from protein phosphatase 2A PR65/A subunit (1B3U) as a template (Groves *et al*., 1999) [56]. The choice of the PR65/A HEAT repeats was based on work by Andrade and colleagues [57], which suggested that the HEAT repeats of Htt belong to the AAA class represented by the PR65/A structure.

## Results

### Polyglutamine expansion of Htt increases its S-nitrosylation

To learn whether cysteine residues of Htt are S-nitrosylated or S-acylated, we expressed both full-length Htt and various Htt fragment constructs in cultured cell lines (Fig 1). The N-terminal fragment (N548, residues 1–548 a.a.) is comparable in size to major *in vivo* cleavage products derived from full-length Htt [15, 58].

These experiments employed a “resin-assisted capture” (RAC) protocol that is based on selective retrieval of proteins from cell lysates using Thiopropyl Sepharose beads that covalently bind to the sulfhydryl groups which were exposed upon reduction of S-nitrosylated cysteine residues (by ascorbic acid) or S-acylated residues (by hydroxylamine). The bound proteins were then eluted with sample buffer containing β-mercaptoethanol and analyzed by Western blotting [52, 53].

**Fig 2:**
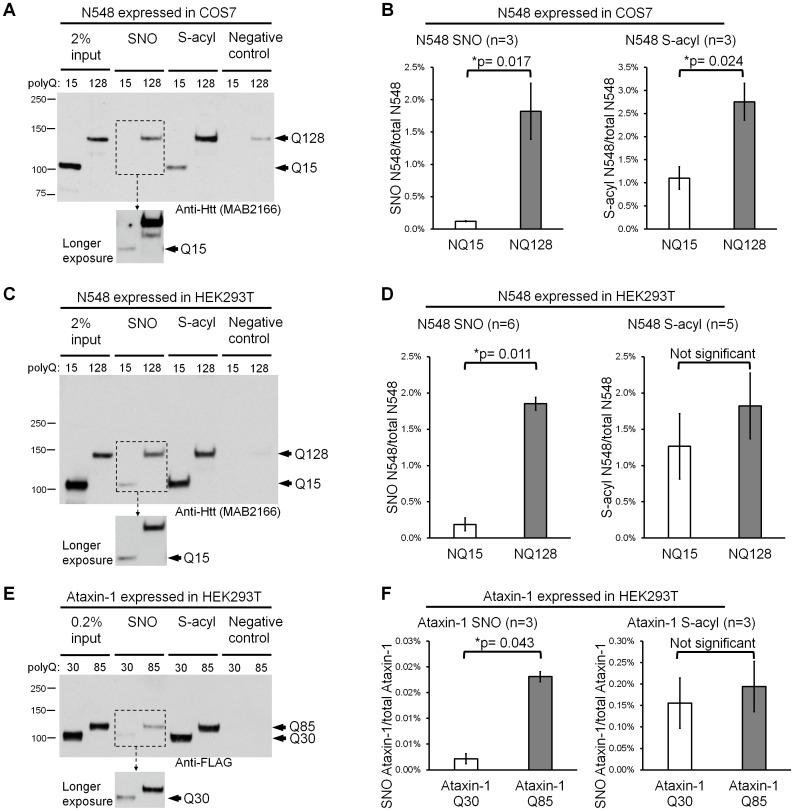
PolyQ expansion increases S-nitrosylation of Htt and Ataxin-1. Recombinant proteins were expressed in cells for one day. SNO-RAC and acyl-RAC were used to recover S-nitrosylated and S-acylated proteins, respectively. The negative control shows that non-specific binding is negligible. Western blotting was used to detect Htt or Ataxin-1 (FLAG-tagged). (A) SNO and S-acylation of Htt N548 fragments expressed in COS7 cells. The longer exposure of the film shows the weak SNO signal from N548 with a normal polyQ tract. (B) Quantification of S-nitrosylated and S-acylated N548 expressed in COS7 cells (n = 3). (C) SNO and S-acylation of Htt N548 fragments expressed in HEK293T cells. (D) Quantitation of S-nitrosylated and S-acylated N548 expressed in HEK293T cells (n = 6 for S-nitrosylation and n = 5 for S-acylation). (E) SNO and S-acylation of Ataxin-1 proteins expressed in HEK293T cells. (F) Quantitation of S-nitrosylated and S-acylated Ataxin-1 expressed in HEK293T cells (n = 3). SNO: S-nitrosylation. S-acyl: S-acylation. ImageJ was used to determine band intensity in all figures. *p*-values from t-test are indicated if p<0.05. Error bars represent SEM in all figures.

After one day transfection of COS7 cells (green monkey) with plasmids encoding N548 Q15 or N548 Q128, the proteins were readily detectable in the respective cell lysates (Fig 2A first two lanes, input). S-nitrosylated and S-acylated Htt were also readily detectable, as judged by Western blotting (Fig 2A, lane 3–6). The extent of both modifications depended on polyQ expansion. Thus, in experiments using two Htt N548 constructs (Q15, Q128) we observed that polyQ expansion was strongly linked to N548 S-nitrosylation (Fig 2A, lane 3 vs 4), increasing Htt SNO levels by ~15 fold (Fig 2B). A more modest increase of S-acylation (~2.5 fold) also accompanied polyglutamine expansion (Fig 2A, lane 5 vs 6, and Fig 2B).

When similar experiments were performed with HEK293T (human) cells, we again detected both modifications and noted that polyQ expansion stimulates S-nitrosylation of Htt much more strongly than S-acylation (Fig 2C). When normalized to total Htt, polyQ expansion increased SNO-Htt ~ 9.8 fold while the impact on S-acylation was not significant (Fig 2D).

A striking increase of S-nitrosylation and a lesser increase of S-acylation was also observed when full-length Htt +/- polyglutamine expansion was expressed in HEK293T cells (S1A and S1B Fig). Thus, in two different host cells, polyglutamine expansion (Q128) consistently caused a major increase in the extent of S-nitrosylation and a lesser degree of S-acylation of Htt (Q128) by comparison to controls (Q15).

### Polyglutamine expansion of Ataxin-1 increases its S-nitrosylation

To learn whether the relation between polyQ expansion and increased S-nitrosylation is specific to Htt, we conducted similar studies of Ataxin-1, which causes spinocerebellar ataxia type 1 (SCA1) when its N-terminal polyQ tract is expanded. For this purpose, we expressed a wild-type control, FLAG-tagged Ataxin-1Q30, or pathogenic Ataxin-1Q85 (S2A and S2B Fig) in HEK293T cells. We again found that polyQ expansion increased S-nitrosylation (Fig 2E and 2F). Increased S-nitrosylation due to polyQ expansion therefore is characteristic of at least two proteins implicated in disease.

### Polyglutamine-dependent high molecular weight species are enriched in Cys-modified forms

The monomeric size of FLAG-Ataxin-1Q30 and FLAG-Ataxin-1Q85, based on protein sequence, are ~88 and ~95 kDa, respectively (Fig 2E). Nevertheless, the MW estimated by gel mobility are ~115 (Q30) and ~130 (Q85) kDa. This deviation due to the polyQ tract is consistent with previous studies [59].

**Fig 3:**
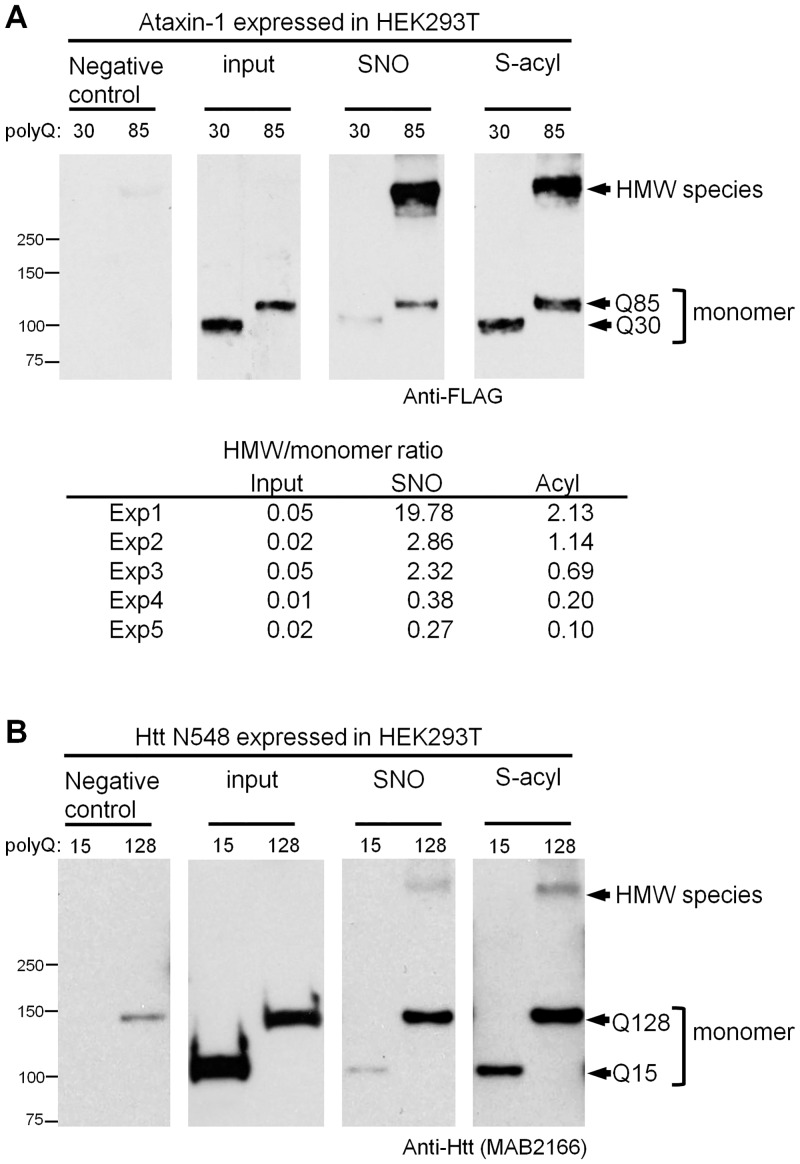
PolyQ expansion in Ataxin-1 and Htt N548 proteins increases S-nitrosylated and S-acylated high molecular weight (HMW) species. SNO-RAC and acyl-RAC were used to recover S-nitrosylated and S-acylated proteins followed by Western blotting. (A) Top: S-nitrosylated and S-acylated monomer and high molecular weight (HMW) species of Ataxin-1 expressed in HEK293T cells. Bottom: HMW/monomer quantity ratios for Ataxin-1Q85 (n = 5). All experiments show that HMW material is enriched in S-nitrosylated and S-acylated proteins. (B) S-nitrosylated and S-acylated monomer and HMW species of Htt N548 expressed in HEK293T cells.

Surprisingly, a high molecular weight (HMW) species ~300 kDa for Ataxin-1Q85 was also seen in the samples that were RAC-enriched either for SNO or for S-acylation. These species were barely observed in the input (Fig 3A). Importantly, such higher molecular bands were not seen in the similarly enriched proteins recovered from cells that express the control, FLAG-Ataxin-1Q30.

PolyQ-dependent HMW species were also observed in extracts of cells expressing Htt N548Q128 when processed by SNO-RAC or acyl-RAC (Fig 3B). The calculated molecular weight of these species is ~290 kDa by comparison to the monomeric band detected by Western blotting (~140 kDa). By comparison to the input panel (Fig 3C), these HMW species were also enriched in the samples recovered by SNO-RAC or acyl-RAC. Nevertheless, the relative titer of HMW forms of N548Q128 was more variable and these forms were much less abundant (by comparison to monomer) than for Ataxin-1Q85.

In all experiments, samples were prepared for SDS-PAGE by addition of β-mercaptoethanol (β-ME) and boiling; however, the HMW material was also detected when reduction by β-ME was at 37°C (S2C Fig). Thus, these HMW species were not an artefact of boiling prior to SDS-PAGE.

### Identification of multiple S-nitrosylation and S-acylation sites in Htt

The primary structure of huntingtin includes several distinct domains (Fig 1A). At the N-terminus upstream of the polyQ tract is a serine-rich phosphorylated short domain (1–17 a.a.) that lacks cysteine residues. This is followed by the polyglutamine-containing segment and a short polyproline tract. After the polyproline region, much of the sequence of Htt can be modeled as being composed of clusters of HEAT motifs, each of which consists of a helix-turn-helix [57]. No specific functions have been attributed to the domains of Htt. *In vivo* cleavage at any of several sites, as indicated in Fig 1, is characteristic of pathogenesis [32].

To locate the cysteine residues that are modified, COS7 and HEK293T cells were transfected to express the N548 N-terminal fragment (Q15 or Q128) of Htt and processed. COS7 cells were of particular interest since they had been used to study S-palmitoylation of Htt [15]. HEK293T cells provide a higher yield of the recombinant proteins and therefore facilitate detection of modified cysteine residues.

To identify the modified cysteine residues, it is necessary to avoid changes of cysteine sulfhydryl status upon cell lysis. For this purpose, the cell lysis buffer included N-ethylmaleimide (NEM). After elimination of excess NEM and reduction with ascorbate to selectively expose the cysteine sulfhydryl groups that had participated in S-nitrosylation, the samples were blocked with iodoacetamide and then immunoprecipitated. In parallel, to detect sites that were S-acylated, samples were reduced with hydroxylamine before iodoacetamide-labeling and immunoprecipitation. Samples were then separated by SDS-PAGE. The bands of purified Htt proteins were recovered for in-gel digestion. Tryptic peptides were then separated and analyzed by liquid chromatography coupled with mass spectrometry.

**Fig 4:**
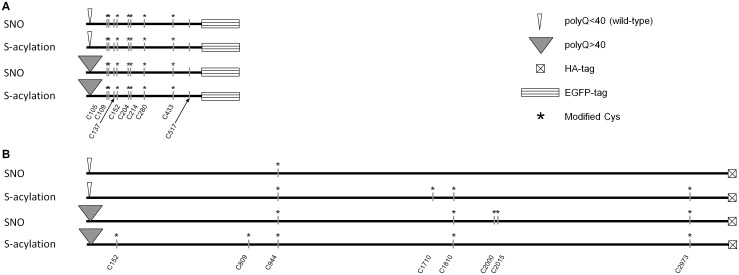
Summary of the SNO and S-acylation sites identified by LC-MS/MS. (A) Htt N548 fragments. Vertical lines indicate all Cys residues in the N548 fragment. Stars label those that were modified. Modifications on C137 and C517 were not detected in three independent experiments. C137 and C517 are indicated by arrows. (B) Full-length Htt. MS-detected modifications of Cys are labeled for full-length Htt.

As summarized in Fig 4A and Table 1, seven of the nine cysteine residues in the N548 fragment were identified and most were detected in both cell types. Moreover, all sites that could be S-nitrosylated were also found to be S-acylated.

**Table 1:** Summary of SNO and S-acylation sites in Htt N548Q15 and N548Q128.

Identified peptide sequence	Site modified by IAA	IAA modified (%)^a^						Fold change (Mean: Q128/Q15)^c^
		Exp 1^b^ (N548)		Exp 2^b^ (N548-C214S)		Exp 3^b^ (N548-C204S)		
		Q15	Q128	Q15	Q128	Q15	Q128	
**MVADE****C****LNK**	C152 (SNO)	1.7	4.1	1.1	1.8	0.26	2.1	4.0
**MVADE****C****LNK**	C152 (S-acyl)	1.3	4.3	1.1	1.7	0.34	1.4	3.0
**TAAGSAVSI****C****QHSR**	C280 (SNO)	3.8	2.8	3.1	5.5	0.91	3.3	1.6
**TAAGSAVSI****C****QHSR**	C280 (S-acyl)	2.4	3	2.9	4.1	0.95	2.2	1.7
**SGSIVELIAGGGSS****C****SPVLSR**	C433 (SNO)	3.7	2.6	1.4	2	1.0	0.96	1.0
**SGSIVELIAGGGSS****C****SPVLSR**	C433 (S-acyl)	3.3	2.2	1.3	1.4	1.4	1.4	0.9
**C****RPYLVNLLPSLTR**^d^	C204 (SNO)	N/A	N/A	0^e^	2.1	N/A	N/A	Infinite^e^
**C****RPYLVNLLPSLTR**^d^	C204 (S-acyl)	N/A	N/A	0.92	1.8	N/A	N/A	2.0
**SRPYLVNLLP****C****LTR**^f^	C214 (SNO)	N/A	N/A	N/A	N/A	1.2	6.1	5.1
**SRPYLVNLLP****C****LTR**^f^	C214 (S-acyl)	N/A	N/A	N/A	N/A	3.8^e^	28.1^e^	7.4^e^
**VNH****C****LTI****C****ENIVAQSVR**^g^	C105/C109 (SNO)	N/A	N/A	0.32	0.97	N/A	N/A	3.0
**VNH****C****LTI****C****ENIVAQSVR**^g^	C105/C109 (S-acyl)	N/A	N/A	0.3	0.78	N/A	N/A	2.6

^a^Cysteine S-nitrosylation (SNO) or S-acylation (S-acylation) was reduced and then modified by IAA. The percentage was calculated as IAA-modified/unmodified peptide.

^b^COS7 cells was used for exp1 and exp2. HEK293T was used for exp3.

^c^Mean of IAA-modified Q128 (%)/IAA-modified (%) Q15.

^d^C214S mutation and modifications on C204 were identified.

^e^Low MS signal. Quantitation may not be accurate.

^f^C204 mutation and modifications on C214 were identified. Low MS signal, identified by MS precursor ion and LC retention time compared to unmodified species.

^g^C105 and C109 are very close. Modifications on both residues were combined to calculate modification percentage.

Residues C204 and C214 were both in the same tryptic peptide, but SNO and S-acylation on these residues could be individually identified on the basis of analysis of corresponding Cys→Ser point mutants (C204S and C214S) that we expressed in the same host cells. Two other sites (C105, C109) were also in a single tryptic peptide. MS detected both single and double modifications of this peptide (Table 1).

By expressing full-length Htt in HEK293T cells, we identified S-nitrosylation and S-acylation of additional sites beyond the N-terminal 548 amino acids. Owing to the large size of Htt, the sequence coverage of the protein was incomplete (44%). Six additional SNO sites and eight additional S-acylation sites beyond N548 were identified by mass spectrometry, as summarized in Fig 4B. Consistently, SNO-RAC and acyl-RAC experiments also detected S-nitrosylation and S-acylation of Htt C-terminal fragments (585–3144 a.a.) expressed in cells (S1C Fig).

### Identification of a major site of S-nitrosylation and S-acylation

Given that there are multiple SNO and S-acylation sites, some residues could be more frequently modified than others. Since S-palmitoylation of Htt on C214 has been reported in COS7 cells [15], we began by evaluating SNO and S-acylation of N548 wild-type versus the N548-C214S mutant using SNO-RAC and acyl-RAC.

**Fig 5:**
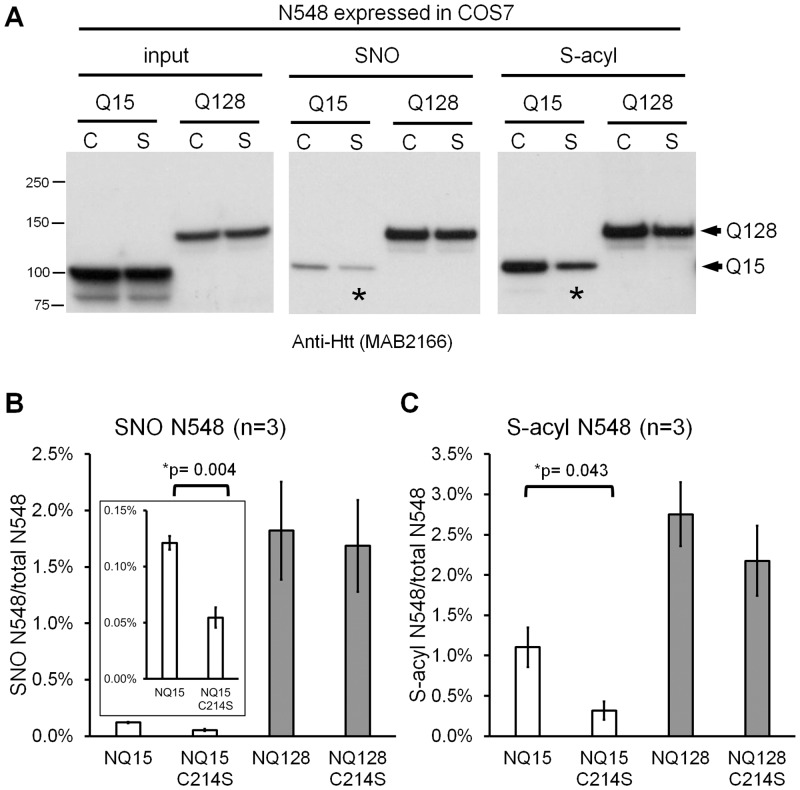
The C214S mutation reduces Htt S-nitrosylation and S-acylation in the context of the normal polyQ tract. (A) S-nitrosylation (SNO) and S-acylation (S-acyl) of Htt N548Q15, N548Q15-C214S, N548Q128, and N548Q128-C214S. Recombinant proteins were expressed in COS7 cells for one day. The stars indicate significant reduction due to the C214S mutation. C: C214; S: C214S. (B) Quantification of the ratio of Htt N548 S-nitrosylation to total N548 (n = 3). The inset enlarges the scale for N548Q15 and N548Q15-C214S. (C) Quantification of the ratio of Htt N548 S-acylation to the total N548 (n = 3). In (B) and (C), experiments for C214 mutants were performed in parallel with Cys wild-type shown in Fig 2A. *p*-values from t-test are indicated if *p*<0.05. The error bar represents the SEM.

In the context of normal polyQ (Htt N548Q15), Htt S-nitrosylation and S-acylation were reduced but not eliminated by this mutation (Fig 5A). SNO was reduced ~50% and S-acylation was reduced ~70% due to this mutation in N548Q15 (Fig 5B and 5C). By contrast, the equivalent mutation had no significant effect on N548Q128. Therefore, the specificity of SNO and S-acylation in Htt appear to be modulated by the length of polyglutamine tract. Regulation of these modifications could contribute to the normal biology of Htt and to pathogenesis.

### Elevated nitric oxide synthase expression increases Htt N548 inclusion formation

**Fig 6:**
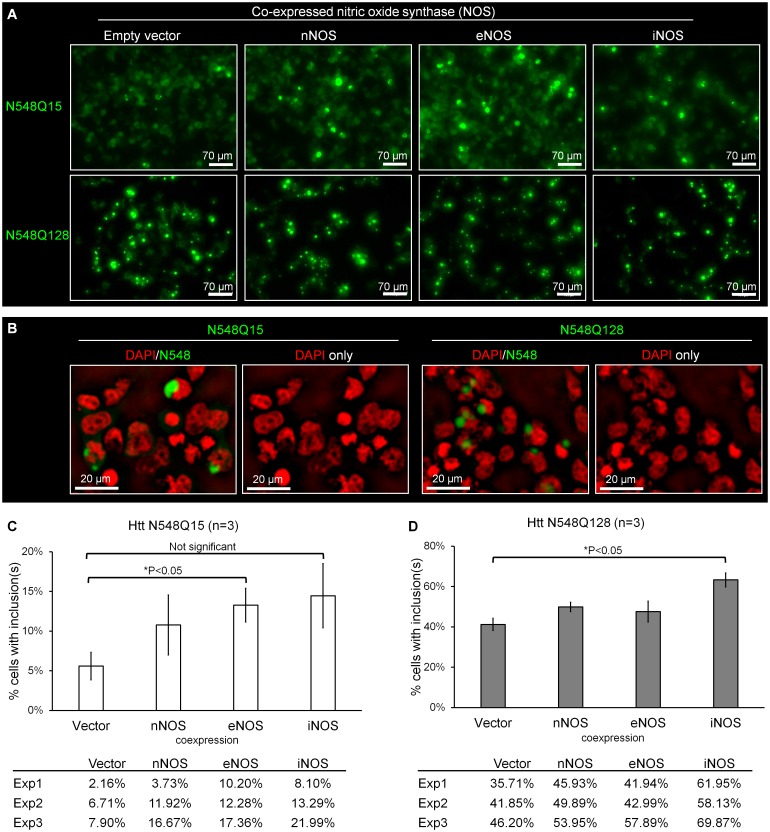
Co-expression of nitric oxide synthase (NOS) promotes Htt N548 inclusion formation. EGFP-tagged Htt N548 fragments were expressed in HEK293T cells for two days. Empty vector co-expression serves as the negative control by comparison to nNOS, eNOS, or iNOS. (A) Representative fluorescence microscopy images of co-expression of NOS and N548 showing the fluorescent inclusions that are present. All images were acquired under the same parameters. (B) Htt N548Q15 and N548Q128 inclusions are adjacent to the nucleus. A single focal plane (2 μm thickness) is shown. The DAPI signal is shown in red and EGFP-tagged N548 signal in green. (C) The percentage of cells with N548Q15 inclusions in the presence of NOS expression. Top: average of three experiments; bottom: individual experiment (n = 3). (D) The percentage of cells with N548Q128 inclusions in the presence of NOS expression (n = 3). Top: average of three experiments; bottom: individual experiments. For quantification, we divided the number of cells with inclusions by the total number of cells expressing EGFP signal. The error bar represents the SEM.

The Htt N548 fragment forms cytoplasmic inclusions in cultured cells in a polyQ-dependent manner [15]. It has been reported that polyQ expansion increases Htt aggregates [5]. We also find that the frequency of inclusions is greater in cells expressing Q128 than for Q15 (Fig 6A). Because polyQ expansion notably increases SNO modification of Htt, nitric oxide synthase (NOS) level could modulate the formation of inclusions. Indeed, we found that co-expression of NOS increased Htt N548 inclusion formation (Fig 6A). Typically, the inclusions are located adjacent to the nucleus (Fig 6B).

In three experiments, cells co-expressing nNOS, eNOS, or iNOS had increased levels of inclusions of N548Q15 and N548Q128 (cells with inclusions/total EGFP-positive cells), as compared to empty vector controls (Fig 6C and 6D). The modest *p*-values for these differences upon nNOS coexpression may be due to the small sample size (n = 3). Statistic “effect size” has been found to be a useful indicator of the treatment effect on the observed outcome [60]. It is not influenced by the sample size. Based on Cohen's effect size standard (expressed as Cohen's *d*; *d* value), *d*> 0.80 indicates a large effect that cannot be neglected. The effect size calculated in this study was *d* = 1.02 for N548Q15 (nNOS coexpression versus vector coexpression control) and *d* = 1.85 for N548Q128 (nNOS coexpression versus vector coexpression control). Therefore the effect of the nNOS expression cannot be neglected.

Although their size and shape were variable, the inclusions in different preparation were similar to each other (S3 Fig). As illustrated, the frequency of inclusion formation (N548Q15 and N548Q128) was increased significantly (*p*<0.05) upon expression of eNOS or iNOS, respectively (Fig 6C and 6D). Specifically, eNOS increased N548Q15 inclusions ~2 fold whereas iNOS increased N548Q128 inclusions to a lesser extent (Fig 6C and 6D). We also detected ~2 fold increase in S-nitrosylated N548Q15 upon co-expression of eNOS or iNOS (S4A–S4C Fig), suggesting that SNO is involved in Htt inclusion formation.

### Polyglutamine expanded Htt does not affect global S-nitrosylation and S-acylation

Since polyQ expansion of Htt increases its S-nitrosylation and S-acylation, we asked whether expression of polyQ-expanded Htt changes SNO and S-acylation of other proteins in the same cells.

In a first approach, we purified the complete S-nitrosylated and S-acylated proteomes of HEK293T cells using SNO-RAC and acyl-RAC. Coomassie blue staining of corresponding gels showed that cells expressing polyQ-expanded Htt did not obviously change the global pattern of S-nitrosylation or S-acylation (S5A Fig).

In a second approach, we asked whether the presence of polyQ-expanded Htt would increase SNO or S-acylation of wild-type Htt. For this purpose, we co-expressed Htt exon1-Q97 along with N548Q15 in HEK293T cells. No remarkable increase of S-nitrosylation or S-acylation of N548Q15 was found in repeated experiments (S5B–S5D Fig). Equivalent results were obtained in experiments with cells that co-express N548Q128 and full-length HttQ15 (S5E–S5G Fig).

The lack of stimulation might reflect the inability of these pairs of recombinant proteins to bind each other. Alternatively, there may be a limit to the amount of SNO or S-acylation that can be accepted by HttQ15. It is also possible that the nitrosylation and acylation machineries act only on abnormally aggregated Htt. Nevertheless, experiments co-expressing nitric oxide synthase (NOS) and the N548 fragment did not show remarkable co-immunoprecipitation of N548 and NOS due to polyQ expansion (S4D and S4E Fig).

## Discussion

Huntingtin is highly conserved among vertebrates and chordates [61] and most of its many cysteine residues are conserved among vertebrates. In human Htt, there are seventy cysteine residues. Htt undergoes both S-nitrosylation and S-acylation and both modifications are increased by polyQ expansion. This relation seems to be of general significance for polyQ-containing proteins since parallel increases are seen for Ataxin-1. Further examples may well be found in additional proteins with polyQ tracts. Judging from the present observations on both the N548 fragment and the C-terminal region (585–3144 a.a.) of Htt, many of the cysteine residues of the full-length protein may actually be modified. These posttranslational modifications would be expected to affect local features of Htt domains and might cause deleterious gain of function. Moreover, their widespread distribution could have broader impact on global properties of Htt.

A fundamental question that emerges from these studies is the mechanism by which polyQ expansion increases cysteine modifications. One might envisage a change in levels of nitric oxide (NO) or of transferases and in fact there has been a report of elevated NO in cells expressing polyQ-expanded Htt [62]. We have observed that recombinant nNOS and eNOS can be co-immunoprecipitated with Htt N548 fragments expressed in HEK293T cells (S4E Fig). Nevertheless, we did not detect a remarkable difference in the degree of NOS association due to polyQ expansion.

Considering that cysteine residues are broadly distributed along the length of the protein (Fig 1), the observation that polyQ expansion has a major effect on the overall level of modification may best be attributed to global conformational features of Htt that alter reactivity of these residues as well as their interactomes, which may include NOSs and nitrosylases [48, 63, 64]. PolyQ expansion may also increase NOS activity in the cytoplasm.

We have in fact noticed one indication of higher-level reorganization of Ataxin-1 and Htt upon polyQ expansion. This is the presence of high molecular weight species that are S-nitrosylated and S-acylated. None of these species is eliminated by reduction with β-ME, suggesting novel chemistry and perhaps linkage to other proteins. A recent report has found that disulfide formation, which may be promoted by S-nitrosylation [65] can mediate Htt oligomerization [66]. The HMW species could correspond to a distinct Htt configuration that is resistant to β-ME.

Our mass spectrometry data identified multiple cysteine residues that are S-nitrosylated. When we align the amino acid sequence of 21-mers centered on these S-nitrosylated residues, we find that seven of nine are flanked by both negatively charged (D/E) and positively charged (K/R/H) residues (S6 Fig), fitting a canonical acid-base motif model [67, 68]. The only exception is C280 (which is also modified), perhaps because of its interaction with other proteins. Our experiments show that C137 and C517 are not S-nitrosylated. Neither of these cysteines is flanked by positively charged residue in the 21-mers. C137 does however lie in a context (I/L-X-C-X_2_-D/E) that is predicted to be nitrosylated by the iNOS/S100A8/S100A9 complex [48]. We do not know whether this complex is formed in COS7 and HEK293T cells. Alternatively, Htt may be regulated by other NOS complexes that do not include S100A8/S100A9. The striking presence of multiple sites in Htt suggests an atypical role for its S-nitrosylation, considering that many S-nitrosylated proteins have no more than a few modified site [69].

There is no *a priori* reason to expect selected residues to be preferentially modified. However, we observe that removal of a single site (C214) has a significant effect on the level of S-nitrosylation and S-acylation of wild-type Htt, but only a modest effect on polyQ expanded Htt. C214 is also a site of Htt S-palmitoylation by HIP14 [15], a palmitoyl acyltransferase enriched in the brain where it colocalizes with the Golgi [70, 71].

Since the chemical properties of NO and acyl moieties are very different in size and hydrophobicity, it is likely that a shift from one to the other will cause changes of spatial relations with nearby residues. These potential interactions could be either *in cis*, between separate copies of Htt, or between Htt and other proteins. In several other proteins, S-nitrosylation and S-acylation also occur on the same cysteine residues or are regulated in reciprocal fashion. Well-established examples concern GAP-43, SNAP-25 and PSD-95. PSD-95 is of central importance for regulation of NMDA receptors and neuronal survival [45, 72, 73].

Many proteins undergo S-nitrosylation or S-acylation but the extent of modification is often unknown. Some of these modifications could regulate specific functions and reactivities, while others could have more global effects on protein structure. In this view, considering the length of huntingtin and the broad distribution of its cysteine residues, its entire structure might undergo repeated fluctuations due to these (and other) modifications. It is also possible that these modifications reflect the age of Htt molecule.

Htt is a defining member of the HEAT motif group of proteins (Htt, Elongation factor 3, protein phosphatase 2A, and the yeast kinase TOR1) [74]. Individual HEAT motifs include a pair of short helices with a Leu-rich hydrophobic core [57]. HEAT repeats form solenoid-like structures that often mediate protein-protein interaction [75]. For example, the HEAT repeats of CRM1/XPO1 (exportin 1) interact with many export cargos, and one cysteine (C528) is critical for these interactions [76–78]. S-nitrosylation of this residue (or alkylation with leptomycin B) abolishes interaction with cargo due to steric interference [76, 77]. We therefore performed computational simulation using the MMM server, based on the prediction of Tartari *et al*. [9] (S7 Fig). The template is the first 8 HEAT repeats of the PR65/A structure (1B3U) since Andrade *et al*. classified Htt HEAT motifs into the group represented by PR65/A [57]. Several clusters of HEAT motifs are found in the Htt sequence [32, 79] (Fig 1).

The N-terminal 548 a.a (N548) fragment on which we have concentrated includes the first cluster (S7 Fig) and the first seven of nine cysteine residues, all of which are conserved and are located in helices. C137, by contrast, is unmodified and is situated in an intervening loop. As shown in S7b Fig, C105, C109, and C152 may be adjacent to each other and therefore collectively influence the structure of the α-helices in which they reside. C204 and C214, on the other hand, are located in neighboring helices and may have their sulfhydryl groups oriented in such a fashion that interactions are unlikely. C280 is further downstream and far-separated from other modified cysteine residues.

In summary, we document a general effect of polyglutamine expansion on protein S-nitrosylation and S-acylation. These changes could modulate protein conformation and the progression of disease. We also observe that Htt inclusions increase in response to NOS overexpression and that the inclusions show an intimate relation to nuclei. Additionally, we found that endogenous Htt is S-nitrosylated in multiple mouse tissues (S1D and S1E Fig). PolyQ-dependent S-nitrosylation is also observed in the PC12 pheochromocytoma cell line (S1F and S1G Fig), which has been used as a neuronal cell model [80]. We therefore hypothesize that polyQ-dependent modifications impact the dynamics of local, and perhaps global protein conformation. On a broader scale, such effects may provide a rationalization for a general function of cysteine residues of proteins in the relatively reducing environment of the cytosol.

## Supporting Information

S1 Fig Full-length Htt S-nitrosylation is polyQ-dependent and the fragment beyond the N-terminal region is modified. (A) PolyQ expansion increases full-length Htt S-nitrosylation. Full-length HttQ15 and HttQ128 were expressed in HEK293T cells for one day. Cell extracts were processed for SNO-RAC and acyl-RAC as in Fig 2. (B) Longer exposure for detecting S-nitrosylated Htt. (C) The Htt C-terminal fragment (C-t 585–3144 a.a.) is S-nitrosylated and S-acylated. Full-length HttQ15 (FL) and the Htt C-terminal fragment were expressed in COS7 cells for one day. Full-length HttQ15 serves as a positive control for S-nitrosylation and S-acylation. (D) Endogenous wild-type Htt in B6 mouse tissues are S-nitrosylated. Samples without ascorbic acid treatment are the negative control for SNO-RAC. (E) Input loading control for endogenous Htt in mouse tissues. Some degradation of full-length Htt was observed even the protease inhibitor cocktail was in the lysis buffer. (F) PolyQ expansion increases full-length Htt S-nitrosylation in PC12 pheochromocytoma cell line. Inducible recombinant HttQ23 and HttQ73 constructs were induced by adding ponasterone A (5 μM) for two days. Samples without ascorbic acid treatment are the negative control. Images of S-nitrosylated HttQ23 and HttQ73 are from the same membrane. (G) Input loading control for recombinant HttQ23 or HttQ73 expressed in PC12 cells. Images of HttQ23 and HttQ73 are from the same membrane. The indistinguishable migration rates of full-length HttQ23 and HttQ73 is due to the relatively small difference in polyQ length (Q23 versus Q73). SNO: S-nitrosylation. S-acyl: S-acylation. SNO-RAC and acyl-RAC were used to recover S-nitrosylated and S-acylated proteins, respectively. The negative control (no ascorbic acid or hydroxylamine reduction) shows non-specific binding is negligible. Western blotting was used to detect Htt. MAB2166 and MAB2050 detect N-terminal and C-terminal regions, respectively.
(TIF)

S2 Fig The high molecular weight (HMW) species of Ataxin-1Q85 do not disappear after 37°C incubation with sample buffer. (A) Diagram of full-length Ataxin-1. The horizontal open boxes indicate the AXH domain, Ataxin-1/HBP1 (HMG box-containing protein 1 transcription factor). AXH of Ataxin-1 is a protein-protein interacting domain (Orr, H.T., *Prog Neurobiol*. 2012). The vertical lines indicates all 6 Cys residues. Cysteine residue numbers are indicated. Polyglutamine is interrupted by His residues. Ataxin-1 with uninterrupted polyQ≥ 39 is pathogenic. (B) FLAG-tagged full-length Ataxin-1 proteins. Q30 and Q85 were used in this study. (C) SNO-RAC and acyl-RAC were performed (3 hr bead binding) to purify S-nitrosylated and S-acylated Ataxin-1Q85. The supernatant of RAC was reserved to run the Western. In parallel, input control was incubated with or without reducer (ascorbic acid or NH2OH) for 3 hr. FLAG-tagged Ataxin-1Q85 was transiently expressed in HET293T cells for 1 day. ASC: ascorbic acid. SNO: S-nitrosylated proteins. S-acyl: S-acylated proteins. Negative control: no reagents to reduce S-nitrosylated/S-acylated proteins for pull-down.
(TIF)

S3 Fig Inclusions of N548-EGFP in cells expressing nitric oxide synthase. Although their size and shape are variable, the inclusions in different preparation are similar to each other. EGFP-tagged Htt N548 fragments were expressed in HEK293T cells for two days. Empty vector co-expression serves as the control for co-expression of nNOS, eNOS, or iNOS. DAPI signal is shown in blue and EGFP-tagged N548 signal in green. All images were acquired under the same parameters. Two fields are illustrated for each condition.
(TIF)

S4 Fig NOS co-expansion increases S-nitrosylation of Htt N548Q15 and NOS-N548 interaction is not significantly affected by polyQ expansion. (A), (B), and (C) NOS overexpression increases Htt N548Q15 S-nitrosylation. Recombinant proteins were expressed in HEK293T cells for one day. Htt N548Q15 was co-expressed with empty vector control (v), nNOS (n), eNOS (e), or iNOS (i). SNO-RAC and acyl-RAC were used to recover S-nitrosylated and S-acylated proteins, respectively. Western blotting was used to detect Htt, nNOS, eNOS, and iNOS. (A) Input loading controls for Htt N548Q15 co-expressed with empty vector (v), nNOS (n), eNOS (e), or iNOS (i). SNO: S-nitrosylation. (B) S-nitrosylation of N548Q15 co-expressed with empty vector control (v), nNOS (n), eNOS (e), or iNOS (i). (C) The quantification of S-nitrosylated N548Q15. SNO N548Q15 content is normalized to input N548Q15. Empty vector co-expression is set to one fold. ImageJ was used to determine band intensity. (D) and (E) PolyQ expansion in Htt N548 fragment does not significantly affect physical association of nitric oxide synthase (NOS) and N548 fragments. Htt N548 fragments were co-expressed with nNOS, eNOS, or iNOS in HEK239T cells for one day. Immunoprecipitation (IP) and Western blotting (WB) were used to detect NOS-Htt interaction. (D) N548 fragments recovered by IP with anti-EGFP. (E) Co-precipitated NOS with N548 fragments. Co-precipitation of nNOS or eNOS was detected whereas coprecipitated iNOS was not detectable. PolyQ expansion in N548 did not significantly change the N548-nNOS or N548-eNOS interaction.
(TIF)

S5 Fig Expression of polyQ-expanded Htt does not significantly increase S-nitrosylation and S-acylation of global proteins and wild-type Htt. (A) Htt expression does not significantly change global S-nitrosylation and S-acylation. Full-length Htt proteins or N548 fragments was expressed in HEK293T cells. Extracted proteins were used for SNO-RAC and acyl-RAC. Purified S-nitrosylated and S-acylated proteomes were detected by Coomassie blue staining. N15: cell expressing N548Q15; N128:N548Q128; NT: no transfection; F15: full-length HttQ15; F128: full-length HttQ128. The band indicated by the star has an expected gel mobility of N548Q128. (B) to (G) PolyQ-expanded Htt does not significantly increase S-nitrosylation and S-acylation of wild-type Htt. Wild-type (normal polyQ<40) Htt protein (full-length or N548) was co-expressed with the polyQ-expanded Htt fragment (N548 or exon1-coding region). For the control samples, wild-type Htt was co-expressed with an empty vector control (v), wild-type N548 or wild-type exon1. SNO-RAC and acyl-RAC followed by Western blotting were used to detect S-nitrosylation and S-acylation of wild-type Htt co-expressed with other constructs. In this experiments, MAB2166 recognizes full-length Htt and N548 but not exon1. MAB2168 recognizes the C-terminal region of full-length Htt but not N548/exon1. N548 and exon1 are EGFP-tagged. (B), (C), and (D) Co-expression of Htt exon1Q97 or N548Q128 does not increase S-nitrosylation and S-acylation of N548Q15 (bands indicated by stars). (B) Input loading control. (C) S-nitrosylation of N548Q15 co-expressed with other constructs. (D) S-acylation of N548Q15 co-expressed with other constructs. (E), (F), and (G) Co-expression of Htt exon1Q97 or N548Q128 does not increase S-nitrosylation and S-acylation of full-length HttQ15 (bands indicated by stars). (E) Input loading control. (F) S-nitrosylation of HttQ15 co-expressed with other constructs. (G) S-acylation of HttQ15 co-expressed with other constructs. SNO: S-nitrosylation. S-acyl: S-acylation.
(TIF)

S6 Fig Flanking sequences of S-nitrosylated cysteine residues of Htt. The 21-mers centered on cysteine residues are presented. The iNOS complex contains iNOS, S100A8, and S100A9 proteins. CRM1 C517 is the SNO and leptomycin alkylation site.
(TIF)

S7 Fig Local environment of S-nitrosylated cysteine residues of Htt. (A) Computer-simulated Htt HEAT repeat cluster 1 (79–397 a.a.). MS-identified S-nitrosylation and S-acylation sites are indicated in red. In three independent experiments, we found no evidence of modification of C137. The sequence between polyQ and Gly79 is the 38-residue long polyproline tract (PPPPPPPPPPPQLPQPPPQAQPLLPQPQPPPPPPPPPP). (B) Enlarged side view of 79–165 a.a. region from (A). Residues beyond Leu165 are masked.
(TIF)

## Abbreviations

HD: Huntington’s disease
Htt: Huntingtin
SCA1: Spinocerebellar ataxia type 1
PolyQ: polyglutamine
SNO: S-nitrosylation
S-acyl: S-acylation
PTM: posttranslational modification
RAC: resin-assisted capture
MS: mass spectrometry
HEAT: (Htt, Elongation factor 3, protein phosphatase 2A, and the yeast kinase TOR1)
NO: nitric oxide
NOS: nitric oxide synthase
