# Supplementary figures and images for: Polyglutamine Tract Expansion Increases S-Nitrosylation of Huntingtin and Ataxin-1
Source: PLoS One. 2016 Sep 22;11(9):e0163359. doi: 10.1371/journal.pone.0163359 (PMC5033456; doi:10.1371/journal.pone.0163359)

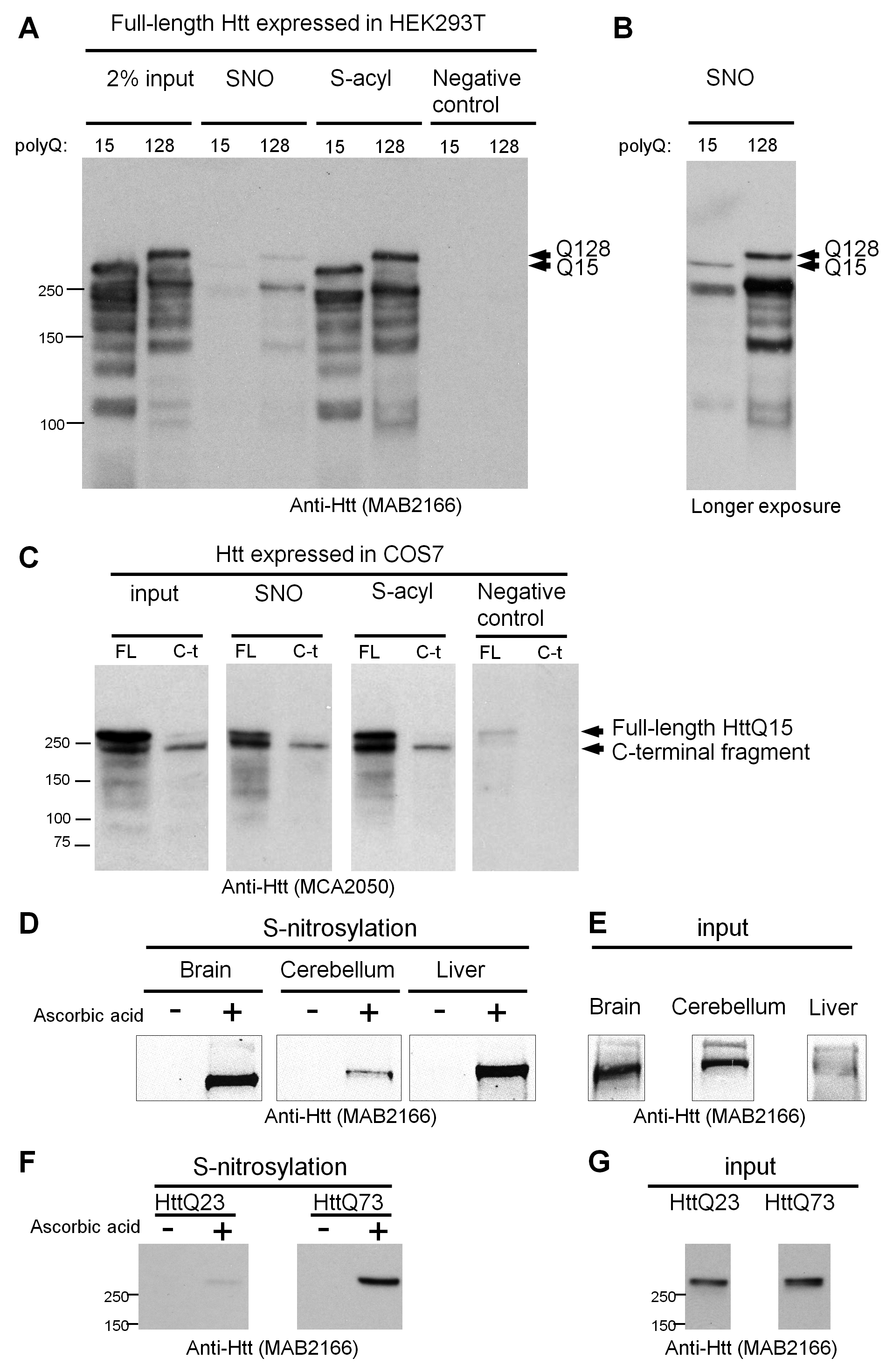

Supplement: S1 Fig — (A) PolyQ expansion increases full-length Htt S-nitrosylation. Full-length HttQ15 and HttQ128 were expressed in HEK293T cells for one day. Cell extracts were processed for SNO-RAC and acyl-RAC as in Fig 2. (B) Longer exposure for detecting S-nitrosylated Htt. (C) The Htt C-terminal fragment (C-t 585–3144 a.a.) is S-nitrosylated and S-acylated. Full-length HttQ15 (FL) and the Htt C-terminal fragment were expressed in COS7 cells for one day. Full-length HttQ15 serves as a positive control for S-nitrosylation and S-acylation. (D) Endogenous wild-type Htt in B6 mouse tissues are S-nitrosylated. Samples without ascorbic acid treatment are the negative control for SNO-RAC. (E) Input loading control for endogenous Htt in mouse tissues. Some degradation of full-length Htt was observed even the protease inhibitor cocktail was in the lysis buffer. (F) PolyQ expansion increases full-length Htt S-nitrosylation in PC12 pheochromocytoma cell line. Inducible recombinant HttQ23 and HttQ73 constructs were induced by adding ponasterone A (5 μM) for two days. Samples without ascorbic acid treatment are the negative control. Images of S-nitrosylated HttQ23 and HttQ73 are from the same membrane. (G) Input loading control for recombinant HttQ23 or HttQ73 expressed in PC12 cells. Images of HttQ23 and HttQ73 are from the same membrane. The indistinguishable migration rates of full-length HttQ23 and HttQ73 is due to the relatively small difference in polyQ length (Q23 versus Q73). SNO: S-nitrosylation. S-acyl: S-acylation. SNO-RAC and acyl-RAC were used to recover S-nitrosylated and S-acylated proteins, respectively. The negative control (no ascorbic acid or hydroxylamine reduction) shows non-specific binding is negligible. Western blotting was used to detect Htt. MAB2166 and MAB2050 detect N-terminal and C-terminal regions, respectively. (TIF) [file pone.0163359.s001.tif]

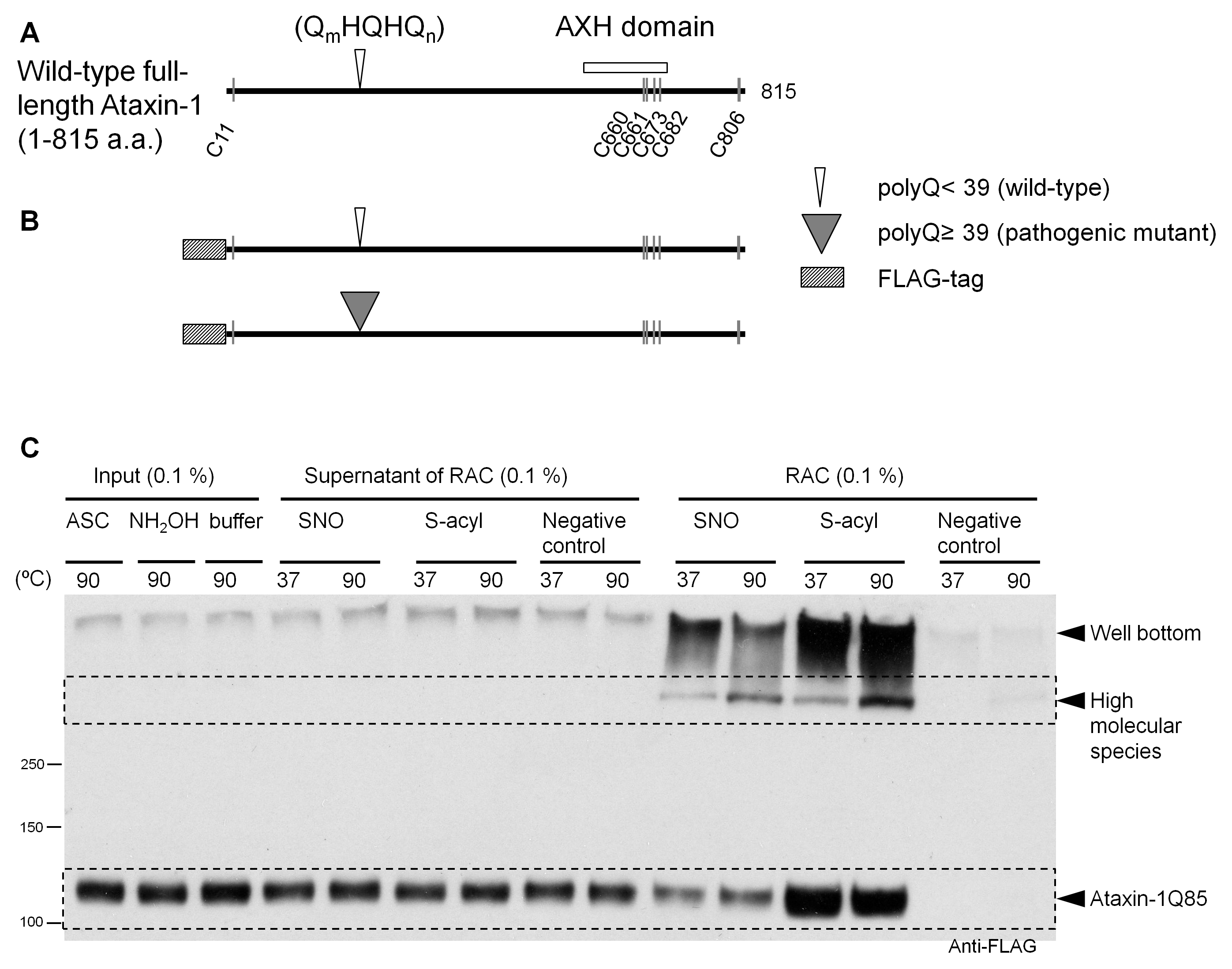

Supplement: S2 Fig — (A) Diagram of full-length Ataxin-1. The horizontal open boxes indicate the AXH domain, Ataxin-1/HBP1 (HMG box-containing protein 1 transcription factor). AXH of Ataxin-1 is a protein-protein interacting domain (Orr, H.T., Prog Neurobiol. 2012). The vertical lines indicates all 6 Cys residues. Cysteine residue numbers are indicated. Polyglutamine is interrupted by His residues. Ataxin-1 with uninterrupted polyQ≥ 39 is pathogenic. (B) FLAG-tagged full-length Ataxin-1 proteins. Q30 and Q85 were used in this study. (C) SNO-RAC and acyl-RAC were performed (3 hr bead binding) to purify S-nitrosylated and S-acylated Ataxin-1Q85. The supernatant of RAC was reserved to run the Western. In parallel, input control was incubated with or without reducer (ascorbic acid or NH2OH) for 3 hr. FLAG-tagged Ataxin-1Q85 was transiently expressed in HET293T cells for 1 day. ASC: ascorbic acid. SNO: S-nitrosylated proteins. S-acyl: S-acylated proteins. Negative control: no reagents to reduce S-nitrosylated/S-acylated proteins for pull-down. (TIF) [file pone.0163359.s002.tif]

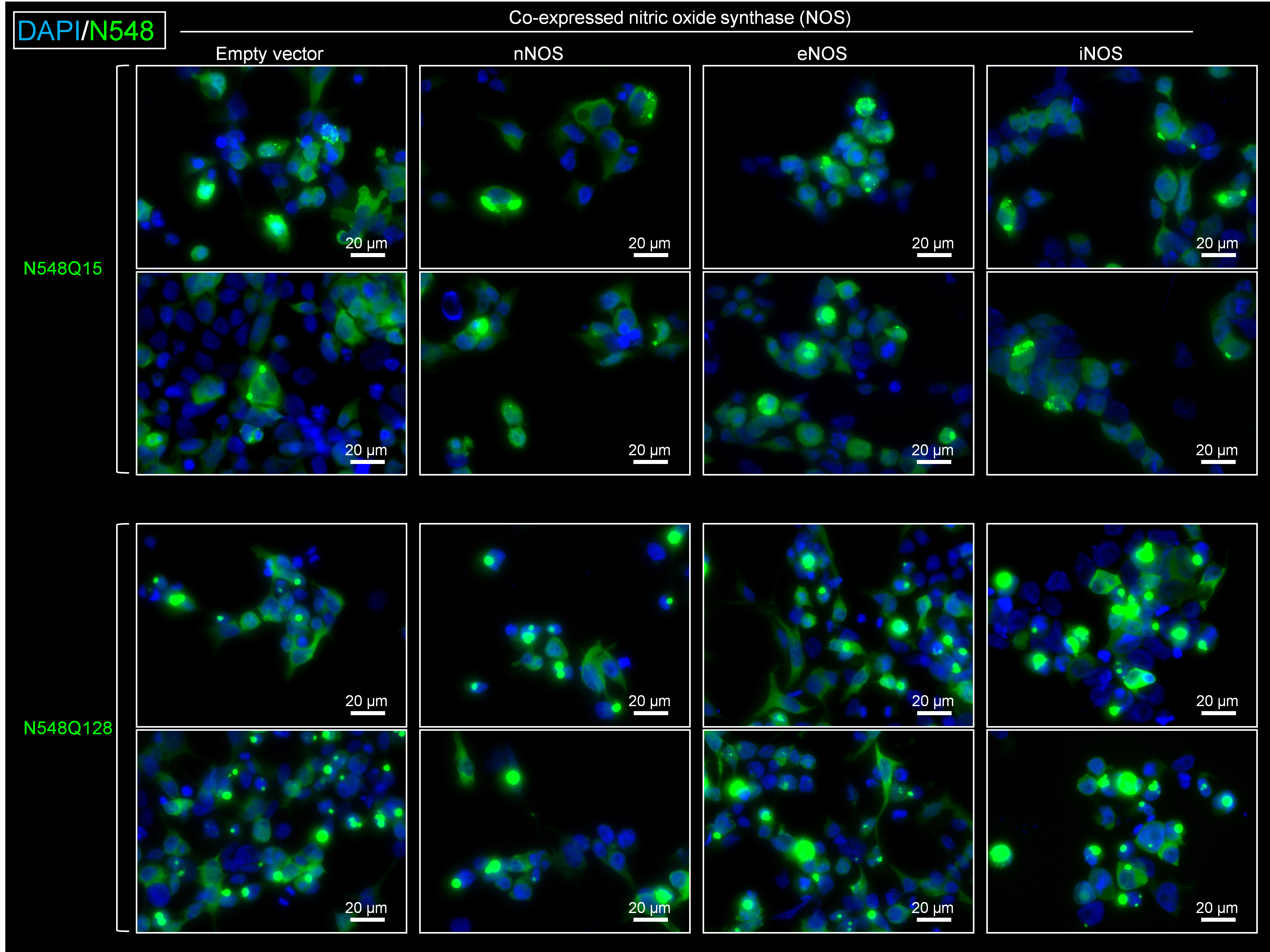

Supplement: S3 Fig — Although their size and shape are variable, the inclusions in different preparation are similar to each other. EGFP-tagged Htt N548 fragments were expressed in HEK293T cells for two days. Empty vector co-expression serves as the control for co-expression of nNOS, eNOS, or iNOS. DAPI signal is shown in blue and EGFP-tagged N548 signal in green. All images were acquired under the same parameters. Two fields are illustrated for each condition. (TIF) [file pone.0163359.s003.tif]

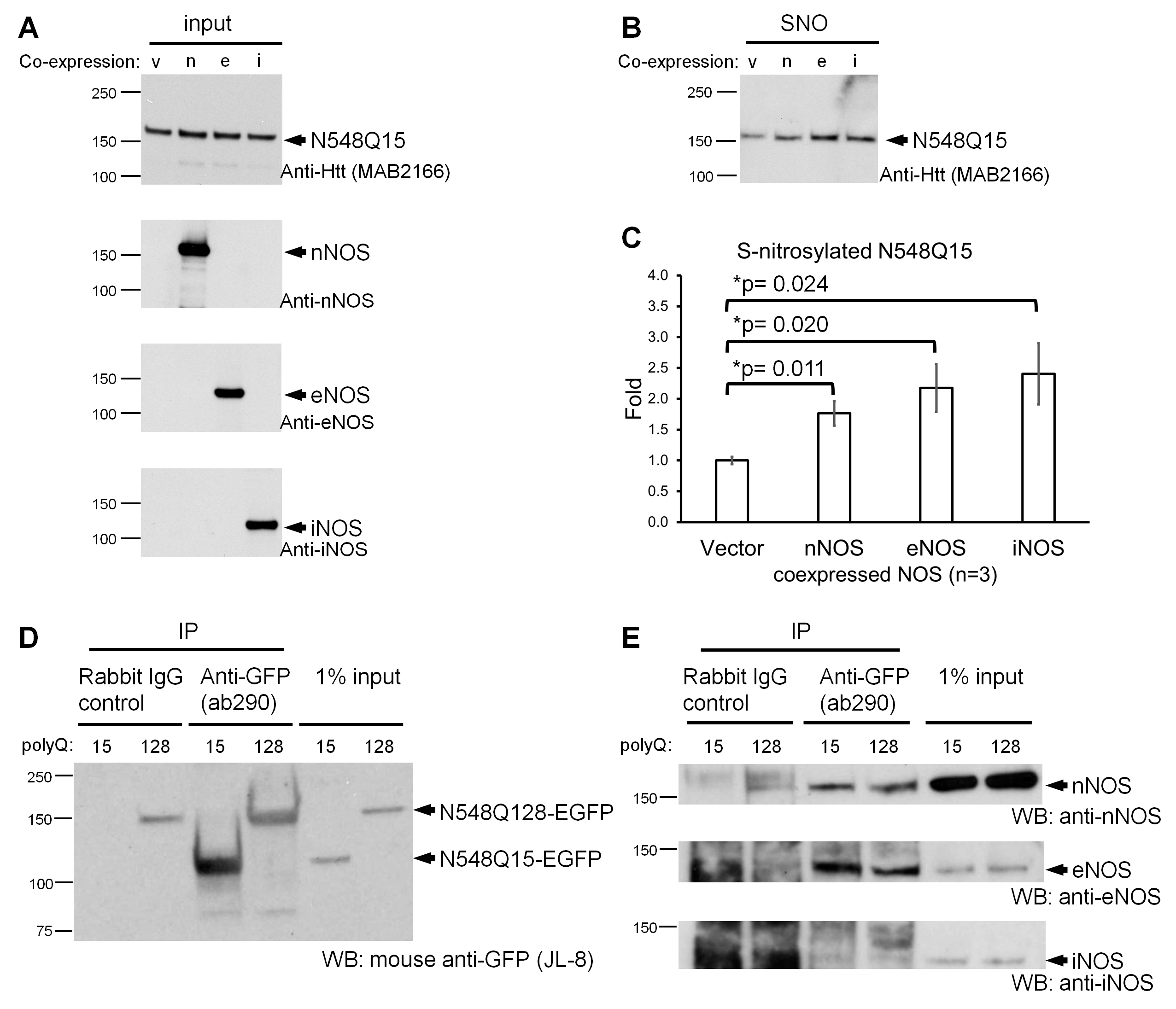

Supplement: S4 Fig — (A), (B), and (C) NOS overexpression increases Htt N548Q15 S-nitrosylation. Recombinant proteins were expressed in HEK293T cells for one day. Htt N548Q15 was co-expressed with empty vector control (v), nNOS (n), eNOS (e), or iNOS (i). SNO-RAC and acyl-RAC were used to recover S-nitrosylated and S-acylated proteins, respectively. Western blotting was used to detect Htt, nNOS, eNOS, and iNOS. (A) Input loading controls for Htt N548Q15 co-expressed with empty vector (v), nNOS (n), eNOS (e), or iNOS (i). SNO: S-nitrosylation. (B) S-nitrosylation of N548Q15 co-expressed with empty vector control (v), nNOS (n), eNOS (e), or iNOS (i). (C) The quantification of S-nitrosylated N548Q15. SNO N548Q15 content is normalized to input N548Q15. Empty vector co-expression is set to one fold. ImageJ was used to determine band intensity. (D) and (E) PolyQ expansion in Htt N548 fragment does not significantly affect physical association of nitric oxide synthase (NOS) and N548 fragments. Htt N548 fragments were co-expressed with nNOS, eNOS, or iNOS in HEK239T cells for one day. Immunoprecipitation (IP) and Western blotting (WB) were used to detect NOS-Htt interaction. (D) N548 fragments recovered by IP with anti-EGFP. (E) Co-precipitated NOS with N548 fragments. Co-precipitation of nNOS or eNOS was detected whereas coprecipitated iNOS was not detectable. PolyQ expansion in N548 did not significantly change the N548-nNOS or N548-eNOS interaction. (TIF) [file pone.0163359.s004.tif]

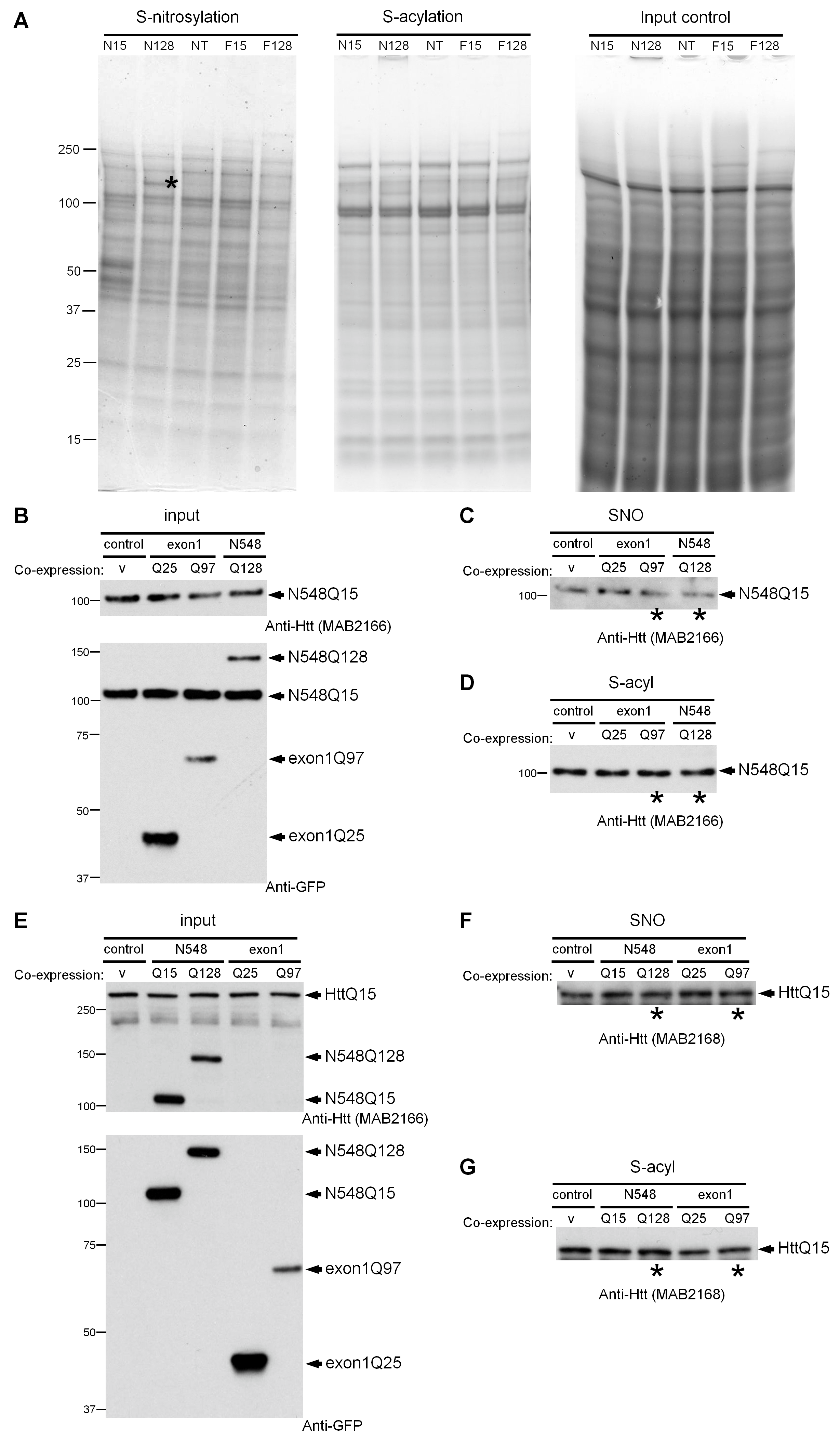

Supplement: S5 Fig — (A) Htt expression does not significantly change global S-nitrosylation and S-acylation. Full-length Htt proteins or N548 fragments was expressed in HEK293T cells. Extracted proteins were used for SNO-RAC and acyl-RAC. Purified S-nitrosylated and S-acylated proteomes were detected by Coomassie blue staining. N15: cell expressing N548Q15; N128:N548Q128; NT: no transfection; F15: full-length HttQ15; F128: full-length HttQ128. The band indicated by the star has an expected gel mobility of N548Q128. (B) to (G) PolyQ-expanded Htt does not significantly increase S-nitrosylation and S-acylation of wild-type Htt. Wild-type (normal polyQ<40) Htt protein (full-length or N548) was co-expressed with the polyQ-expanded Htt fragment (N548 or exon1-coding region). For the control samples, wild-type Htt was co-expressed with an empty vector control (v), wild-type N548 or wild-type exon1. SNO-RAC and acyl-RAC followed by Western blotting were used to detect S-nitrosylation and S-acylation of wild-type Htt co-expressed with other constructs. In this experiments, MAB2166 recognizes full-length Htt and N548 but not exon1. MAB2168 recognizes the C-terminal region of full-length Htt but not N548/exon1. N548 and exon1 are EGFP-tagged. (B), (C), and (D) Co-expression of Htt exon1Q97 or N548Q128 does not increase S-nitrosylation and S-acylation of N548Q15 (bands indicated by stars). (B) Input loading control. (C) S-nitrosylation of N548Q15 co-expressed with other constructs. (D) S-acylation of N548Q15 co-expressed with other constructs. (E), (F), and (G) Co-expression of Htt exon1Q97 or N548Q128 does not increase S-nitrosylation and S-acylation of full-length HttQ15 (bands indicated by stars). (E) Input loading control. (F) S-nitrosylation of HttQ15 co-expressed with other constructs. (G) S-acylation of HttQ15 co-expressed with other constructs. SNO: S-nitrosylation. S-acyl: S-acylation. (TIF) [file pone.0163359.s005.tif]

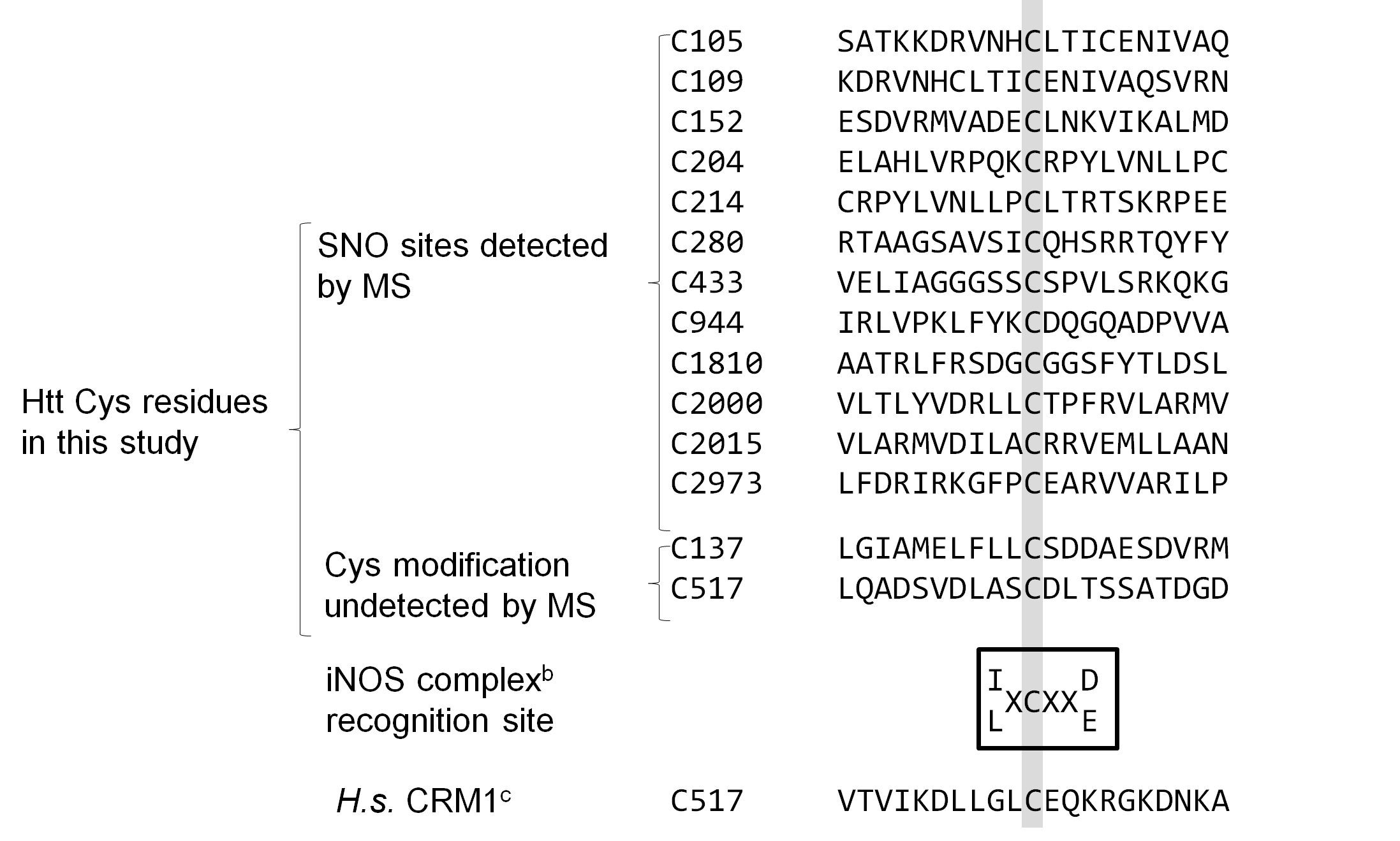

Supplement: S6 Fig — The 21-mers centered on cysteine residues are presented. The iNOS complex contains iNOS, S100A8, and S100A9 proteins. CRM1 C517 is the SNO and leptomycin alkylation site. (TIF) [file pone.0163359.s006.tif]

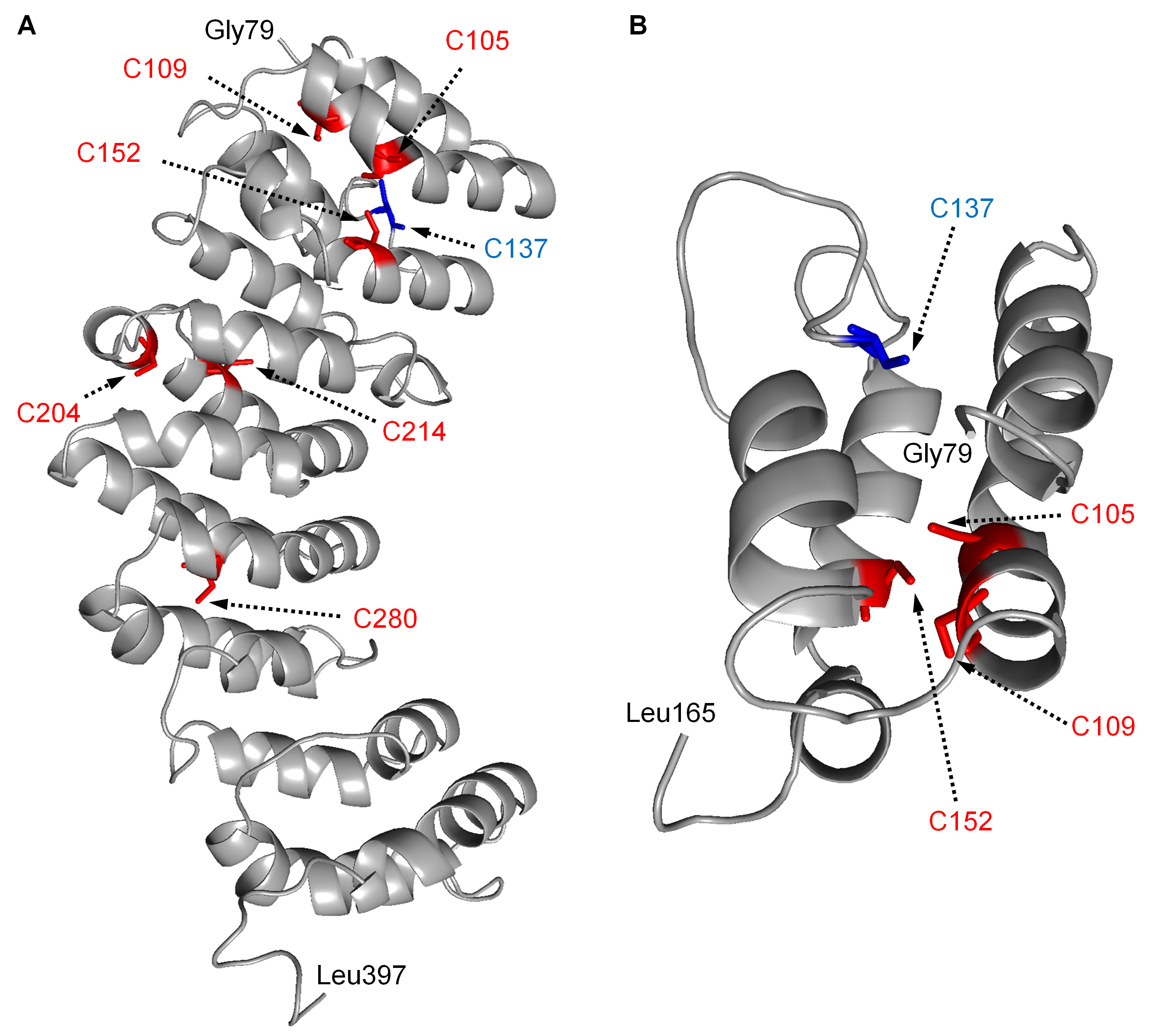

Supplement: S7 Fig — (A) Computer-simulated Htt HEAT repeat cluster 1 (79–397 a.a.). MS-identified S-nitrosylation and S-acylation sites are indicated in red. In three independent experiments, we found no evidence of modification of C137. The sequence between polyQ and Gly79 is the 38-residue long polyproline tract (PPPPPPPPPPPQLPQPPPQAQPLLPQPQPPPPPPPPPP). (B) Enlarged side view of 79–165 a.a. region from (A). Residues beyond Leu165 are masked. (TIF) [file pone.0163359.s007.tif]
